# Supplementary material for: Systemic transphobia and ongoing barriers to healthcare for transgender and nonbinary people: A historical analysis of #TransHealthFail
Source: PLOS Digit Health. 2025 Mar 12;4(3):e0000718. doi: 10.1371/journal.pdig.0000718 (PMC11902059; doi:10.1371/journal.pdig.0000718)
Supplement: Attachment — Submitted filename: ResponseToJournalAndEditorFeedback.docx [file pdig.0000718.s001.docx]

Journal Requirements:

*Since your data is not available for proprietary reasons, please explain via email why the data is not available. Please also include the contact information for the third-party organization that should be contacted should other researchers want to request access to this data and please include the full citation of where the data can be found. We also request that you verify with us via email that any researcher will be able to obtain the data set in the same manner that you have obtained it. If you feel you are unwilling or unable to adhere to this policy, please explain your reasons by return email and your exemption request will be escalated to the editor for approval. Your exemption request will be handled independently and will not hold up the peer review process but will need to be resolved should your manuscript be accepted for publication. One of the Editorial team will be in touch if they require more information.*

**This response has been provided previously by email as request, but we include it here as well as considerable time has passed since the email was sent (in February).**

**The data were obtained by requesting access to Twitter’s Academic Researcher API. The agreement between Twitter and the PI in that case does not allow the person who has received permission to access the data to share the date with others. Any researcher can apply for their own access to the data to enable a historical/archival search as we have performed. However, several changes have been implemented at Twitter (now, X) since we collected these data that might make it more difficult for others to obtain the data. First, data are no longer free to access and cost to access data ranges from $1000 - $5,000USD/mo (value updated 7/2024), which may be cost prohibitive. They have also banned the use of third-party apps to access data (e.g., Mozdeh, which we used). Thus, directly accessing data in the API requires more programming knowledge.**

Editor Feedback and Responses

*This is a timely study which focuses on a topic at the intersection of social, ethical, and public health domains. There are very many results and conclusions here that have high value and pertinence to society as a whole. These strengths are worth exploring and do fit into the scope and mission of the journal, but there are also important matters that should be addressed before going to full review.*

**We appreciate that you can see the potential value of the work and the important points you have raised. Below, we will address each of the key points as well as how we have addressed them.**

*The technique of utilizing a social media hashtag to pull a diverse and accessible range of experiences and stories seemed relatively novel and comprehensive in an unconventional way. One key limitation of this technique for such a dynamic and emotionally charged topic is that, while comments themselves were included for the years 2015-2021, there was not description or limit described for when those experiences actually occurred. For instance, it seems like experiences could have been described from forty years ago when Real Life Experience was mandatory for securing gender affirming medical care and were aggregated with experiences from as recently as four years ago. The WPATH standards of care, society guidelines, the political landscape, and cultural acceptance have vastly changed in content and awareness during these decades. When did the experiences labelled as "Many reported that lack of health insurance coverage, which included blanket exclusions for GATs, often deeming them cosmetic or elective" occur in relation to Section 1557 of the Affordable Care Act? This limitation should be addressed or otherwise conclusions should be substantially tempered with this in mind.*

**Your point is well taken, and we appreciate that you have raised this concern. We have added the lack of timing available for when the described experiences took place in the limitation section. We have also highlighted some of the important changes that have taken place regarding gender affirming care in the last decade, including the reinstatement of the interpretation of ‘sex’ to include gender identity in section 1557 of the ACA, as well as ongoing challenges in the current sociopolitical context.**

*While there is no word count limit on manuscripts, the length related to diffusion of content is an issue. In the materials and methods/data analysis section, four categories are described: antecedents, barriers, outcomes, and consequences. But then in the results not only is a fifth category added, but one of those categories is further broadened into seven more subcategories with 3-5 described items within each of the twelve categories. Then an additional section of more categories continues to expand at the end. This will be overwhelming to reviewers and readers and gives the sense that not enough distillation was performed.*

*There also is currently no way of identifying which categories were more prevalent than others without an idea of the objective distribution of comments into each category. The reader must presume that each issue raised carried equal importance and prevalence to all others.*

- The omission of the category healthcare experiences, which was central to the analysis, was an error in the first draft.
- We have consolidated some of the additions (exacerbating factors is now under barriers and T+ patient strategies are included in the conceptual model as theme that cuts across all of the others).
- The refined conceptual diagram helps to demonstrate the relationship between larger categories and the themes within them.
- We reduced the number of sub-themes that were included, limiting to only those that appeared 20 times or more.
- We also added Table 1, which includes representative quotes for each of the themes (and select sub-themes) within each category. Table 1 also includes some indication of how frequently each of the topics arose. In addition, because Table 1 is helpful at demonstrating the types of content that were organized in each category, we have reduced the amount of description in the results section of the manuscript.

*With any restructuring, if the information is available then there may also be value in delineating a bit more some of the experiences and reports of non-binary transgender people from binary transgender people who may have different concerns from each other.*

**We agree that it could be valuable to delineate the experiences and reports of nonbinary trans folks from those with more binary trans identities. Unfortunately, data on gender identity are not available in the data. In the discussion, we have included this information in the limitations, highlighted what is known about nonbinary people’s healthcare experiences and proposed further research is needed.**

*Another option may include describing each group a bit more before going into the details of types of mistreatment. For instance, some binary trans men and women do not prefer to have pronouns frequently asked and may have had different "hoops" to jump through in demonstrating their new gender role. In contrast some non-binary transgender people may have frustratingly had to sell the "trapped in the wrong body and/or interest in a specific trajectory of medical transition" narrative which binary transgender people might be more comfortable with describing.*

**In reviewing the tweets, there were T+ individuals with a binary gender identity who did not have the experience of being aware of being a different gender at a very early age, feel ‘trapped’ in the wrong body, and/or did not desire a specific medical trajectory. As such, suggesting that this might be a barrier for only nonbinary people was incongruous with these data. We did, however, highlight one of the unique experiences of invalidation that nonbinary T+ folks may experience based on these data, and as noted above, provide additional nuances specific to nonbinary people in the discussion, including the limitation of these data.**

*Lastly there is a surplus of emotionally charged language including alleged legal culpability that should be re-examined before going to review. Examples include "the emergency medical technician stopped treating her when her transgender status became apparent and used derogatory language," "after being refused treatment by more than 20 doctors; by the time someone was willing to treat him, it was too late," "Places who should know better/places that should do better," "T+ care being seen as specialty care, requiring higher copayments," "Often HCP's justification for delaying care was arbitrary," "HCPs also sexualized T+ clients by lewd looks and making remarks about clients' attractiveness," and the use of the term "administrative violence." There is no doubt that the two cases above are great tragedies, but significant care should be made when commenting on legal conclusions related to criminal negligence by remaining as objective as possible. If including these cases is absolutely necessary to frame the study, then some ways around this may be to use terms that suggest possible culpability or reporting conclusions already made in a court of law rather than using language that commands it.*

*For this and many reasons, some verbatim comments from the participants would be valuable to include where pertinent in the results.*

**We appreciate these points and acknowledge that some of the language may be emotionally charged. The references for Tyra Hunter’s case were from reports on the situation and subsequent legal proceedings. We have, however, chosen to remove the content about both Ms. Hunter and Mr. Eads from the introduction. We have also edited much of the other language as recommended.**

**At the same time, we believe other language here is warranted. The phrase “administrative violence” is one that has been used previously in the literature and that is consistent with what folks described in their tweets. Similarly, some individuals did describe being sexualized by their healthcare provider, and we felt it appropriate to acknowledge those experiences. This is another area where the quotes provided in Table 1 could be helpful.**

*There are other notes which reviewers may raise that may be worth investigating before resubmission but are not barriers to going to review. Without being able to read any of the statements objectively written, then it is hard to know if categories such as "comparing lab and test values to the wrong reference range" and "receiving the wrong medication" are related to patient perception or true medical error. For instance, a trans woman might believe that the creatinine range for non-transgender women should be used for herself in all cases and as such report this using the hashtag. But for a variety of physiologic reasons the UCSF Guidelines recommend using the upper limit of the non-transgender male value for creatinine, hemoglobin, and alkaline phosphatase. Were these potential discrepancies taken into account before coding this category? Other statements in a similar vein include "possibly contraindicated medications," "lower quality care," "not receiving accurate information or assessments," and "leaving ER visits without receiving adequate testing." By whose judgment were these conclusions made?*

**Thank you for these points. We have tempered this language to clarify that the tweets suggest people felt they were being treated differently than other people might be. To your point about reference ranges, we have no idea to know whether T+ folks are aware of these standards and, as such, removed the sub-theme of ‘wrong reference range’ from the codes all together. Further, we have included examples of T+ patients’ perceptions of inaccurate education, incorrect referrals, etc. in Table 1.**

*The authors might take care not to communicate with language that overly commodifies healthcare as well.*

**We have replaced the word ‘client’ with ‘patient’ throughout the manuscript.**

*The study reports on a highly important topic and includes valuable results for the community at large. This feedback is not meant to be a discouragement, but hopefully can refine the manuscript in a way that it can reach the most people in the most effective way.*

**Thank you again for your feedback and we believe we have addressed the major concerns that you have raised. We look forward to the opportunity to receive feedback from additional reviewers as well.**
